# Sample size calculation practices in original research articles of the Journal of Conservative Dentistry and Endodontics (2021–2025)

**DOI:** 10.4317/jced.63814

**Published:** 2026-02-26

**Authors:** Marco Sánchez-Tito

**Affiliations:** 1 Research Group on Dental Biomaterials and Natural Products, Faculty of Health Sciences, Universidad Privada de Tacna, 23000 Tacna, Peru

## Abstract

**Background:**

Sample size calculation is essential for methodological rigor and statistical validity in dental research, yet deficiencies in its reporting and coherence with statistical analysis remain frequent. This study aimed to evaluate the frequency and methodological coherence of sample size calculation practices in original research articles published in the Journal of Conservative Dentistry and Endodontics (JCDE).

**Material and Methods:**

A methodological review was conducted, including original research articles published between January 2021 and December 2025. Extracted variables comprised study design, sample size reporting, characteristics of the sample size calculation, statistical tests applied, and coherence between the calculation model and the final analysis. Study designs were grouped into in vitro, clinical, and observational categories. Descriptive statistics were summarized using absolute and relative frequencies. Bivariate associations were assessed using the chi-square test, and multivariable logistic regression was used to identify factors independently associated with methodological coherence.

**Results:**

Of 631 eligible articles, 176 (27.9%) reported a sample size calculation. Complete and methodologically coherent reporting was identified in only 9 studies (1.43%). Methodological coherence differed significantly by study design in bivariate analyses (p &lt; 0.05). In the adjusted model, clinical and observational studies showed higher odds of coherent sample size calculation compared with in vitro studies.

**Conclusions:**

Sample size calculations in JCDE are infrequently reported and often methodologically incoherent. Study design appears to be a key determinant of coherence, underscoring the need for improved integration of statistical planning and reporting standards.

## Introduction

Sample size calculation is a fundamental component of robust research design in the biomedical sciences, as it directly affects statistical power, precision, and the validity of study conclusions (1 , 2). Adequate sample size estimation enables the detection of clinically or experimentally meaningful effects while minimizing the risk of type I and type II errors, thereby strengthening the internal validity and reproducibility of research findings. This aspect is particularly relevant in dentistry, where in vitro studies are widely used to evaluate material properties and physicochemical or mechanical outcomes, often under controlled experimental conditions that demand rigorous methodological justification. Despite its importance, methodological reviews in biomedical and dental research have consistently reported deficiencies in the reporting and application of sample size calculations (3 - 8). Common problems include omission of sample size estimation or incomplete reporting of key parameters such as effect size, variance assumptions, significance level, and target statistical power (2 , 5). In dental research, especially in in vitro studies, sample sizes are frequently determined by convenience rather than formal calculation, even when established methods exist for commonly applied statistical analyses such as ANOVA or repeated-measures designs (6). In addition, sample size calculations are often based on simple two-group comparisons, whereas the final analyses involve multifactorial or multivariate models, introducing a mismatch between the estimated and actual analytical frameworks, and increasing the risk of underpowered or methodologically inconsistent studies (1 , 7). Within this context, the Journal of Conservative Dentistry and Endodontics (JCDE) represents a suitable source for methodological appraisal. It is the official journal of the Indian Association of Conservative Dentistry and Endodontics and has been indexed in PubMed since 2010, publishing a substantial volume of original research in conservative dentistry, dental materials, and endodontics. Recent bibliometric analyses have highlighted its increasing international visibility and methodological diversity (9 , 10), making it a relevant platform for examining contemporary research practices in this field, and supporting its relevance for evaluating current sample size calculation practices. Nevertheless, despite the availability of reporting guidelines that emphasize transparent and coherent justification of sample size estimation (11), adherence in dental journals remains inconsistent. This gap underscores the need for focused methodological evaluations at the journal level. Accordingly, the present study aimed to evaluate the frequency, reporting characteristics, and methodological coherence of sample size calculation practices in original research articles published in the JCDE over the last five years, and to examine their alignment with the statistical analyses applied.

## Material and Methods

A methodological review with descriptive and inferential components was conducted to evaluate the reporting quality and methodological coherence of sample size calculation in original research articles published in the JCDE over a five-year period (January 2021-December 2025). Articles were identified through the official journal website (https://journals.lww.com/jcde) and cross-checked in PubMed to ensure completeness. All original research articles published in all regular issues of the journal available up to December 2025 were included; no partially released or incomplete issues were considered. Only articles classified as original research were included; editorials, reviews, case reports or case series, letters to the editor, and other non-original article types were excluded. For each eligible article, the following data were extracted: year and month of publication, country of the first author, study design, reported final sample size, and whether a formal sample size calculation was reported. When present, detailed information regarding the sample size calculation was collected, including the statistical test or test family on which the calculation was based, the reported effect size and metric, the significance level (), the planned statistical power, post hoc power when applicable, and the software used. For the purposes of this review, a sample size calculation was considered adequate when it reported, at minimum, the underlying test (or test family), the assumed effect size (with its metric), the level, and the target power, with sufficient detail to allow reproducibility (e.g., software or formula and required inputs). In addition, the primary statistical test applied in the final analysis, the software used for statistical analysis, and the methodological coherence between the sample size calculation and the statistical analysis performed were documented. Methodological coherence was defined as consistency between the statistical model assumed during sample size estimation and the primary inferential analysis used, considering the study design and analytical structure. Data extraction and methodological assessment were performed by a reviewer with formal training and specialization in biostatistics and research methodology, following predefined and standardized decision criteria. To assess intra-rater reliability, 10% of the included articles were randomly selected using the random number generator in Microsoft Excel and independently re-evaluated after a two-week interval. Agreement between both evaluations for all categorical variables was quantified using Cohen's kappa coefficient, yielding a value of 1.00, indicating perfect consistency in data extraction and methodological classification. Data analysis was performed using Stata version 19.0 (StataCorp LLC, College Station, TX, USA). Descriptive statistics were used to summarize study characteristics and reporting practices using absolute and relative frequencies. For inferential analyses, study designs were grouped into in vitro (including laboratory-based and computational models), clinical (randomized clinical trials and preclinical studies), and observational designs (including tomography analyses, surveys, and ex vivo studies), reflecting distinct sample size frameworks. Methodological coherence between the sample size calculation and the statistical analysis was defined a priori as the primary outcome. Bivariate associations were assessed using the chi-square test. Multivariable logistic regression was performed to identify factors independently associated with methodological coherence, with study design as the main predictor. Models were adjusted for year of publication to account for temporal trends. Inferential test complexity was included to reflect differences in analytical requirements influencing sample size planning and was categorized as simple (two-group comparisons and non-parametric equivalents), multigroup (e.g. ANOVA or Kruskal-Wallis), or complex (e.g. repeated-measures, mixed, or multilevel models), based on the primary statistical test reported. The use of sample size calculation software was included as an indicator of formal statistical planning and was classified as dedicated when tools specifically developed for a priori sample size estimation were reported, and as not reported when no software was explicitly stated. Results from logistic regression were reported as odds ratios (ORs) with 95% confidence intervals (CIs), and statistical significance was set at = 0.05 (two-sided). Graphical representations of key findings were generated using GraphPad Prism (GraphPad Software, San Diego, CA, USA).

## Results

**Figure 1:**
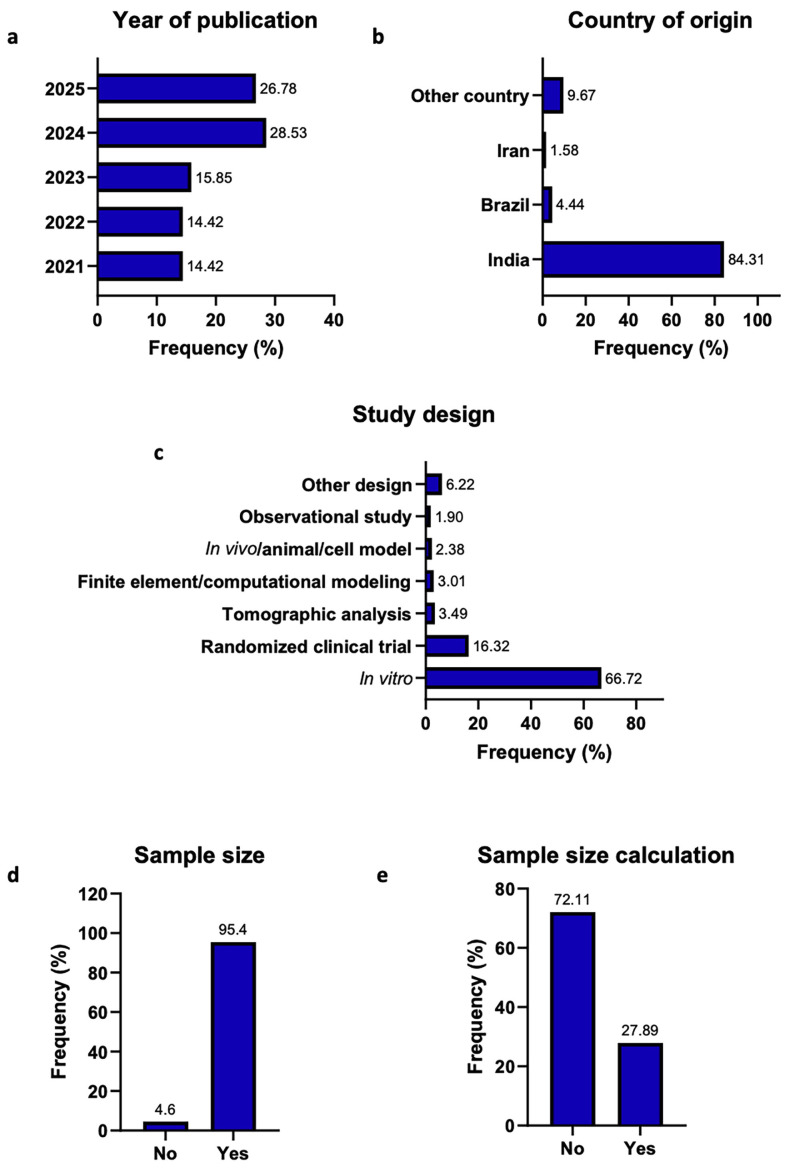
General characteristics of the included original research articles (n = 631). (a) year of publication; (b) country of origin of the first author; (c) study design; (d) Sample size reported; (e) Sample size calculation reported.

Figure 1 summarizes the main characteristics of the 631 original research articles published between 2021 and 2025 included in the analysis.

1 Screenshot

**Figure 2:**
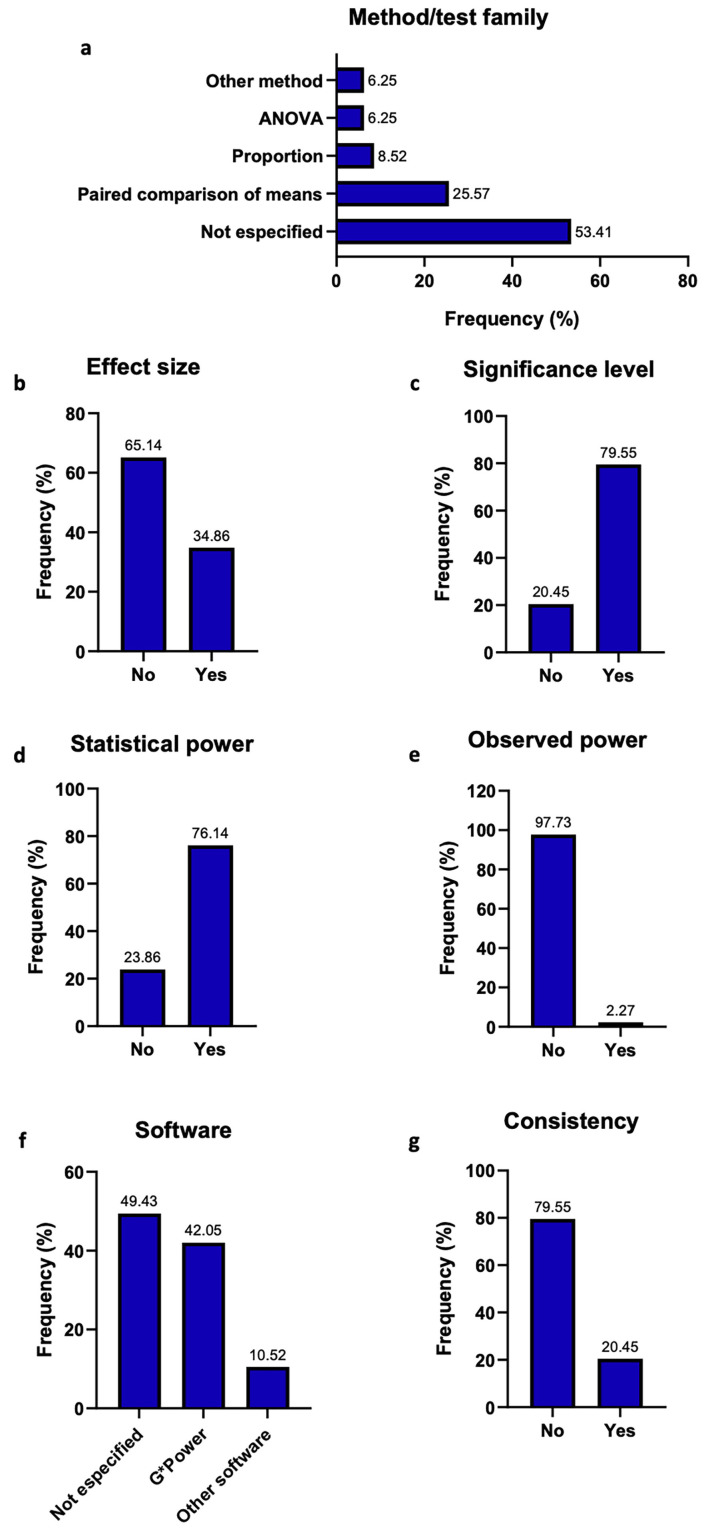
Reported characteristics of studies that performed sample size calculation (n = 176). (a) method or test family used for sample size calculation; (b) frequency of reported effect size for sample size calculation; (c) frequency of reported alpha level for sample size calculation; (d) frequency of reported statistical power for sample size calculation; (e) reported observed (actual) power achieved by the sample size calculation; (f) software used for sample size calculation; and (g) consistency between the sample size calculation and the statistical test applied.

Most publications were concentrated in 2024 and 2025, with India being the predominant country of origin of first authors. In vitro studies constituted the most frequent study design (66.72%). Although sample size was reported in most articles, fewer than one-third included a formal sample size calculation (27.89%), highlighting a notable discrepancy between sample size reporting and appropriate sample size planning. Marked heterogeneity was observed among the 176 studies that reported a sample size calculation. Frequent omissions were noted for key elements such as the calculation method and effect size, whereas the significance level () and planned statistical power were more commonly reported. G*Power was the most frequently identified software, and inconsistencies between the sample size calculation and the statistical test applied remained evident (Fig. 2).

2 Screenshot

Table 1 presents the studies with complete and methodologically coherent reporting of sample size calculation parameters.

Table 1

These articles accounted for only 1.43% of all included studies (9 of 631) and 5.11% of those reporting a sample size calculation (9 of 176), indicating that fully adequate reporting was uncommon even among studies that attempted sample size estimation. Table 2 provides illustrative examples of studies in which inconsistencies or limitations were identified in the reporting of sample size calculations.

Table 2

Across different study designs and analytical approaches, recurrent mismatches were observed between the methods used for sample size estimation and the statistical analyses ultimately performed. Using the chi-square test for bivariate analysis, methodological coherence differed significantly according to study design (p = 0.003) and inferential test complexity (p = 0.022). Higher proportions of coherent reporting were observed in clinical and observational studies compared with in vitro studies, as well as in studies employing simpler inferential tests. No significant associations were identified with year of publication (p = 0.142) or with the use of dedicated sample size calculation software (p = 0.414) (Table 3).

Table 3

Table 4 shows the results of the multivariable logistic regression analysis adjusted for study design, year of publication, sample size calculation software, and inferential test complexity.

Table 4

After adjustment, study design remained independently associated with methodological coherence. Clinical studies showed higher odds of coherent sample size calculation compared with in vitro studies (OR = 3.06; 95% CI: 1.14-8.21; p = 0.026), while observational studies demonstrated an even stronger association (OR = 5.14; 95% CI: 1.36-19.36; p = 0.015). Year of publication, use of dedicated sample size calculation software, and inferential test complexity were not independently associated with methodological coherence in the adjusted model (Table 4).

## Discussion

This methodological review evaluated the reporting quality and methodological coherence of sample size calculations in original research published in the JCDE. The results demonstrate that, although final sample size was routinely reported, formal and prospectively planned sample size calculations were uncommon and rarely methodologically coherent. This pattern indicates that sample size determination is frequently treated as a post hoc formality rather than as an integral component of study design, reflecting a predominantly retrospective rather than prospective approach to statistical planning. Among studies that reported a sample size calculation, incomplete specification of its core components was a recurrent limitation. Effect size was most frequently omitted or only vaguely justified, whereas -level and target power were more consistently reported. This imbalance is problematic because effect size is the primary determinant of required sample size and directly reflects the scientific or clinical relevance of the hypothesized effect. Without explicit and reproducible effect size assumptions, sample size calculations cannot be independently verified or meaningfully interpreted, as previously documented in biomedical and dental research (2 , 5). Furthermore, poorly justified sample sizes substantially weaken the interpretation of nonsignificant findings, since it becomes impossible to distinguish between a true absence of effect and insufficient statistical power (1 , 3). A particularly important finding was the widespread incoherence between the statistical model used for sample size estimation and the statistical analyses ultimately applied. In many studies, sample size calculations were based on simple two-group comparisons, while the final analysis involved multifactorial ANOVA, repeated-measures designs, or longitudinal models. Similar discrepancies have been reported in previous methodological evaluations and reflect a fundamental disconnect between experimental planning and analytical strategy (7 , 8). Because factorial and repeated-measures designs require larger sample sizes to detect interaction effects and account for correlated observations, using calculations based on simpler models systematically underestimates the true sample size required, thereby increasing the risk of false-negative findings and unstable effect estimates. Inferential analyses further demonstrated that these methodological limitations were not uniformly distributed across study types. In the bivariate analysis, methodological coherence differed significantly according to study design and inferential test complexity, with clinical and observational studies exhibiting higher proportions of coherent sample size planning than in vitro studies. This finding suggests that studies involving human participants or observational designs may be subject to greater methodological scrutiny during planning and reporting, whereas experimental in vitro studies appear more prone to simplified or inconsistent sample size justification. The patterns observed across individual articles indicate that these inconsistencies are not isolated reporting errors but represent systematic weaknesses in statistical planning. Common deficiencies included the use of a single sample size to justify multiple endpoints, failure to specify the exact ANOVA design in software-based calculations, and neglect of within-specimen correlation in repeated-measures studies. These practices are well recognized sources of methodological bias and cannot be corrected by post hoc statistical adjustments once data collection has been completed (1 , 4 , 40 - 42). Consequently, they directly compromise both internal validity and reproducibility. Importantly, multivariable logistic regression analysis showed that study design remained independently associated with methodological coherence after adjustment for year of publication, inferential test complexity, and use of dedicated sample size calculation software. Clinical and observational studies showed significantly higher odds of coherent sample size calculation compared with in vitro studies, whereas neither publication year nor use of dedicated software retained an independent association. These results indicate that methodological coherence is more strongly related to the underlying study design than to temporal trends or the mere use of calculation software, underscoring that appropriate statistical planning depends primarily on conceptual alignment between design and analysis rather than on tools alone. Notably, only a small subset of studies fulfilled all fundamental methodological requirements for sample size calculation. The works by Baumeier et al. (12), De Souza et al. (13), Kazemi-Yazdi et al. (14), Elhaddad et al. (15), Kalantri et al. (16), Hendam et al. (17), Yassa et al. (18), Bhattacharyya et al. (19), and Arrué et al. (20) demonstrated complete methodological coherence by clearly specifying the statistical test family, providing justified effect size estimates, using standard and power levels, and aligning the calculation model with the final inferential analysis. The fact that these rigorous examples originated from diverse geographical and research contexts, including in vitro studies from Brazil and Iran and clinical trials from Egypt and India, confirms that high-quality statistical planning is feasible across settings, yet remains the exception rather than the norm within recent JCDE publications. From a methodological and editorial perspective, these findings are especially relevant given current initiatives to strengthen transparency in experimental dental research. The CRIS Guidelines explicitly require clear and reproducible reporting of sample size justification as a prerequisite for the validity of in vitro studies (11). Although both general and dentistry-specific reporting standards are widely available (43 , 44), the present results indicate a persistent gap between these recommendations and actual reporting practices in conservative dentistry and endodontics. This study has certain limitations that should be acknowledged. The analysis was restricted to a single journal, which limits the generalizability of the findings and may partly reflect journal-specific editorial policies rather than broader author behavior. Nevertheless, this focused approach enabled a detailed and internally consistent appraisal of reporting practices within a defined editorial context. In addition, although the study primarily adopted a descriptive methodological framework, the incorporation of inferential analyses aimed to explore systematic differences in coherence across study designs rather than to evaluate the correctness of individual sample size assumptions in depth. The classification of methodological coherence required structured judgement; however, this process was guided by explicit operational definitions and demonstrated perfect intra-rater reliability, minimizing subjectivity. Finally, grouping study designs was necessary to ensure analytical feasibility and model stability, although this may have obscured nuances among specific experimental models with distinct sample size requirements. From a practical standpoint, these results support the need for stricter editorial and peer-review standards regarding sample size reporting. Explicit specification of effect size assumptions, coherent alignment between calculation models and final analyses, and routine use of statistical reporting guidelines would substantially improve the methodological quality, reproducibility, and interpretability of future research in conservative dentistry and endodontics.

## Conclusions

The findings of this methodological review indicate that, although sample size is frequently reported in dental research, formally planned and methodologically coherent sample size calculations remain uncommon. The observed inconsistencies between sample size estimation models and the statistical analyses applied underscore the importance of better integration of statistical planning within study design. Enhancing transparency and coherence in the reporting of sample size calculations may support improvements in methodological rigor, reproducibility, and interpretability of both experimental and epidemiological research in conservative dentistry and endodontics.

## Figures and Tables

**Table 1:** Detailed characteristics of studies with adequate sample size calculation reporting.

Study	Year	Study design	Country of thefirstauthor	Test family used for sample size calculation	Effect size	Method used to estimate effect size	Significance level/ Statistical power	Software used for sample size calculation	Main inferential statistical test
Baumeier et al. [12]	2022	In vitro	Brazil	One-way ANOVA	0.5	Pilot study	5%/80%	G*Power	One-way ANOVA
De Souza et al. [13]	2023	RCT	Brazil	Chi-square test	0.5	Not specified	5%/75%	G*Power	Chi-squared test
Kazemi-Yazdi et al. [14]	2023	In vitro	Iran	One-way ANOVA	0.6	Previous study	5%/80%	PASS	One-way ANOVA
Elhaddad et al. [15]	2024	RCT	Egypt	Chi-square test	0.7	Previous study	5%/80%	G*Power	Chi-squared Test
Kalantri et al. [16]	2024	RCT	India	Proportion comparison	-	-	5%/80%	Formula reported	Chi-squared Test
Hendam et al. [17]	2024	RCT	Egypt	Chi-square test	0.87	Previous study	5%/80%	G*Power	Chi-squared Test
Yassa et al. [18]	2025	In vitro	Egypt	t test	0.12	Previous study	5%/80%	G*Power	Student t test
Bhattacharyya et al. [19]	2025	RCT	India	One-way ANOV A	0.2	Previous study	5%/80%	G*Power	One-way ANOVA
Arrué et al. [20]	2025	In vitro	Brazil	One-way ANOVA	0.92	Previous study	5%/80%	Not declared	One-way ANOVA

1

**Table 2:** Studies with identified inconsistencies or limitations in sample size calculation reporting.

Study	Year	Study design	Country of the first author	Comments
Prado et al. [21]	2021	In vitro	Brazil	The sample size calculation was insufficiently specified, as it was based on a simple ANOVA with an assumed effect size of 1 and did not account for the factorial structure of the final 4 × 4 ANOVA.
Saklecha et al. [22]	2022	RCT	India	The sample size calculation was non-reproducible because the test family was not specified, despite the subsequent use of independent and paired t-tests.
Singla et al. [23]	2023	RCT	India	The sample size calculation did not specify the test family used in G*Power, despite the subsequent use of multiple statistical tests, thereby limiting reproducibility.
Karunakar et al. [24]	2024	RCT	India	The sample size calculation was inconsistent with the statistical analysis, as the ANOVA design was not specified and the subsequent use of repeated-measures ANOVA was not reflected in the estimation, limiting reproducibility.
Sayed et al. [25]	2024	Tomographic analysis	India	Although the authors stated that the sample size was based on means, the formula and inputs corresponded to a two-proportion calculation, indicating a mismatch between the described method and the actual computation.
Gowda et al. [26]	2024	In vitro	India	The sample size calculation did not specify the test used in G*Power and did not account for the separate use of paired and unpaired t-tests in the analysis, limiting reproducibility and methodological consistency.
Saha et al. [27]	2024	In vivo/ex vivo	India	The sample size calculation did not align with the analyses performed, as it was based on repeated-measures ANOVA while paired t-tests, chi-square tests, and Bland–Altman analysis were applied.
Mendonça et al. [28]	2024	Micro-computed tomography	Brazil	The sample size calculation lacked transparency because the effect size for the ANOVA was not reported, preventing reproducibility despite reference to a previous study.
Sharma et al. [29]	2024	RCT	India	The sample size calculation was inconsistent with a four-group design, as a two-mean comparison formula was used and a non-standard parameter (τ) was introduced without specifying a formal multiple-comparison alpha adjustment or a justified primary contrast.
Patel et al. [30]	2024	RCT	India	The sample size calculation was methodologically inconsistent, as a two-group formula was used despite a longitudinal design, and the primary outcome and time point defining the expected difference were not specified, limiting reproducibility.
Chhabra et al. [31]	2024	In vitro	India	The sample size calculation was inconsistent with the analysis, as it assumed a one-way fixed-effects ANOVA while the data were analyzed using a one-way repeated-measures ANOVA across time points.
Saba et al. [32]	2025	In vitro	India	The sample size calculation was insufficiently detailed, as the underlying test and effect size metric were not specified despite the use of ANOVA in the analysis, limiting reproducibility and methodological consistency.
Matallana et al. [33]	2025	In vitro	Brazil	The sample size calculation was inconsistent with the analyses performed, as the ANOVA design used in G*Power was not specified while the data were analyzed using paired and unpaired t-tests and repeated-measures ANOVA. In addition, a single sample size was derived despite multiple reported effect sizes, limiting transparency and reproducibility.
Halkai et al. [34]	2025	In vitro	India	The effect size used for the sample size calculation (0.55) was not justified or linked to a specific outcome, limiting the reproducibility of the calculation.
Krishnegowda et al. [35]	2025	In vitro	India	The sample size calculation was inconsistent with the reported statistical analyses. While the calculation was based on a two-group comparison of means, the analysis involved multiple inter- and intragroup comparisons using ANOVA and post hoc tests.
Shetty et al. [36]	2025	Cross-sectional	India	The sample size calculation was inconsistent with the analysis, as it was based on a two-group mean comparison while the data were analyzed using ANOVA with multiple inter- and intragroup comparisons and post hoc testing.
Rao et al. [37]	2025	Cross-sectional	India	The sample size calculation was misaligned with the reported outcomes, as it was based on prevalence estimation but the results presented only proportional distributions without an overall prevalence estimate or confidence intervals.
Komireddy et al. [38]	2025	In vitro	India	The sample size calculation did not specify the ANOVA design or effect size used, making it difficult to verify its consistency with the one-way ANOVA and Tukey tests reported in the analysis.
Aucancela et al. [39]	2025	In vitro	Ecuador	No formal sample size calculation was reported, and the sample size was arbitrarily defined despite the use of inferential analyses such as chi-square tests and logistic regression without a priori justification of sample adequacy.

2

**Table 3:** Characteristics associated with methodological coherence of sample size calculation in the bivariate analysis (n = 176).

Variable	Coherence of sample size calculation		p-value*
	No (n=140)	Yes (n=36)	
Study design			0.003
In vitro	86 (88.66)	(11 (11.34)	
Clinical	43 (70.49)	18 (29.51)	
Observational	11 (61.11)	7 (38.89)	
Year of publication			0.142
2021	16 (72.73)	6 (27.27)	
2022	12 (66.67)	6 (33.33)	
2023	21 (84.00)	4 (16.00)	
2024	41 (74.55)	14 (25.45)	
2025	50 (89.29)	6 (10.71)	
Software of sample calculation			0.414
Not reported	71 (77.17)	21 (22.83)	
Dedicated software	69 (82.14)	15 (17.86)	
Inferential test complexity			0.022
Simple	56 (70.00)	24 (30.00)	
Multigroup	65 (87.84)	9 (12.16)	
Complex	11 (84.62)	2 (15.38)	

* Chi-square test

**Table 4:** Association between study design and methodological coherence of sample size calculation.

Variable	Bivariate analysis			Multiple regression*		
	OR	95% CI	p-value	OR	95% CI	p-value
Study design						
In vitro	Ref.			Ref.		
Clinical	3.27	1.42 – 7.54	0.005	3.06	1.14 – 8.21	0.026
Observational	4.97	1.59 – 15.50	0.006	5.14	1.36 – 19.36	0.015
Year of publication						
2021	Ref.			Ref.		
2022	1.33	0.34 – 5.17	0.678	1.46	0.30 – 7.17	0.634
2023	0.50	0.12 – 2.10	0.351	0.41	0.08 – 2.02	0.278
2024	0.91	0.29 – 2.78	0.869	0.75	0.20 – 2.74	0.673
2025	0.32	0.09 – 1.13	0.077	0.31	0.07 – 1.34	0.118
Software of sample calculation						
Not reported	Ref.			Ref.		
Dedicated software	0.73	0.35 – 1.54	0.415	0.79	0.34 – 1.86	0.602
Inferential test complexity						
Simple	Ref.			Ref.		
Multigroup	0.32	0.13 – 0.75	0.009	0.65	0.23 – 1.79	0.408
Complex	0.42	0.08 – 2.06	0.288	0.65	0.10 – 3.90	0.641

*Adjusted for study design, year of publication, sample size calculation software, and inferential test complexity. OR: Odds ratio. 95% CI: 95% confidence interval.

## Data Availability

The datasets used and/or analyzed during the current study are available from the corresponding author.
